# SGLT2 Inhibitors and Sleep Quality in Heart Failure: Implications for Patient‐Centered Outcomes

**DOI:** 10.1002/clc.70208

**Published:** 2025-09-09

**Authors:** Surender Himral, Jaibharat Sharma, Shivali Sandal, Kunal Mahajan

**Affiliations:** ^1^ Himachal Heart Institute Mandi Himachal Pradesh India

Dear Editor,

1

We read with great interest the article by Erbay et al. [1] evaluating the impact of sodium‐glucose co‐transporter‐2 (SGLT2) inhibitors on sleep quality, anxiety, and quality of life in patients with heart failure (HF). The study contributes meaningfully to the dialog surrounding patient‐centered outcomes in HF management. However, several important methodological limitations essential for interpretation were not sufficiently discussed in the manuscript. First, the nonrandomized, physician‐directed allocation of SGLT2 inhibitor therapy in this study introduces a substantial potential for selection bias and confounding by indication. Physicians may preferentially prescribe SGLT2 inhibitors to patients who are perceived to be more likely to benefit, be clinically stable, or demonstrate better adherence [2]. Such confounding can extend beyond the measured baseline parameters and affect both clinical and subjective outcomes, including sleep and anxiety. Second, the study groups were imbalanced with respect to key baseline characteristics: the SGLT2 cohort was notably younger and had a significantly higher prevalence of diabetes mellitus (DM). Both age and DM status independently alter sleep architecture, anxiety, and overall quality of life, increasing the risk that these factors, rather than the intervention itself, underpin the observed improvements [3]. In small sample sizes, the capacity to statistically adjust for these complex interrelations is inherently limited. Third, sleep quality assessment in the study relied solely on the Pittsburgh Sleep Quality Index (PSQI), a validated but subjective questionnaire, without incorporating objective sleep measures such as actigraphy or polysomnography. Given the high prevalence of undiagnosed sleep‐disordered breathing in HF and the recognized limitations of self‐reported measures in this population, the absence of objective evaluation may introduce measurement bias and limit causal inference [4]. Fourth, the study is limited by its short follow‐up duration (6 months) and the absence of granular data on changes in concurrent HF and psychotropic medications during follow‐up. Short‐term improvements may not be sustained over time, and undocumented modifications in concomitant therapy can confound the attribution of the observed benefits exclusively to SGLT2 inhibitors. Furthermore, differential attrition, particularly resulting from hospitalization or mortality, exacerbates the potential for survivor bias [5], a critical consideration in the HF population. Taken together, these limitations underscore the need for cautious interpretation of the reported benefits and reinforce the imperative for future randomized, controlled, and objectively assessed studies with longer follow‐ups to establish the patient‐centered efficacy of SGLT2 inhibitors in HF.

## Author Contributions

All authors contributed to the writing of the correspondence and have approved the final version.

## Ethics Statement

The authors have nothing to report.

## Conflicts of Interest

The authors declare no conflicts of interest.

## Data Availability

Data sharing is not applicable to this article as no data sets were generated or analyzed during the current study.
